# Ecofriendly synthesized Zeolite 4A for the treatment of a multi-cationic contaminant-based effluent: Central composite design (CCD) statistical approach

**DOI:** 10.1016/j.heliyon.2024.e35176

**Published:** 2024-07-30

**Authors:** Joseph M. Nseke, Nomsa P. Baloyi

**Affiliations:** Mineral Processing and Technology Research Centre, Department of Metallurgy, School of Mining, Metallurgy and Chemical Engineering, Faculty of Engineering and the Built Environment, University of Johannesburg, P.O. Box 524, Auckland Park 2006, Johannesburg, South Africa

**Keywords:** Ecofriendly synthesized Zeolite 4A, Central composite design, Multi-cationic contaminant, Effluent

## Abstract

One of the key aspects of futureproofing the sustainability of life on earth lies in the protection of the hydrosphere, particularly from soluble heavy metal ion pollutants. In the current study, the central composite design and optimization of the ion-exchange process have been carried out for the simultaneous removal of selected cations; Cd^2+^, Cu^2+^, and Zn^2+^ cations using synthesized zeolite 4A. X-ray diffraction analysis confirmed the formation of zeolite 4A. The Brunauer-Emmett-Teller (BET) surface area of the synthesized zeolite was 32 m^2^/g. Results mainly indicate that there is a strong relationship between the experimental data and central composite design-based models of ion removal efficiency with R^2^ > 0.9 and the lack of fit less than 0.1 %. All the selected ion exchange parameters (time, dosage, pH, and temperature) were found to be statistically significant, with a p-value less than 0.05.

For the complete simultaneous removal of selected cations, the optimal zeolite dosage, pH, and contact time are 1.2 g/100 cm^3^, 6, and 3 h. The optimal temperature ranges from 25 to 27 °C. The initial concentration of each selected cation is 450 mg/L. The ion exchange is in good agreement with the Freundlich and Langmuir isotherm models. Based on the Langmuir isotherm model, the maximum Cd^2+^, Cu^2+^, and Zn^2+^ uptake capacity values of zeolite are 103, 99.89, and 82.08 mg/g, respectively. In this study, it has been mainly inferred that CCD can be considered a useful tool for the modeling and optimization of zeolite ion exchange applications.

## Graphical abstract

**Figure:**
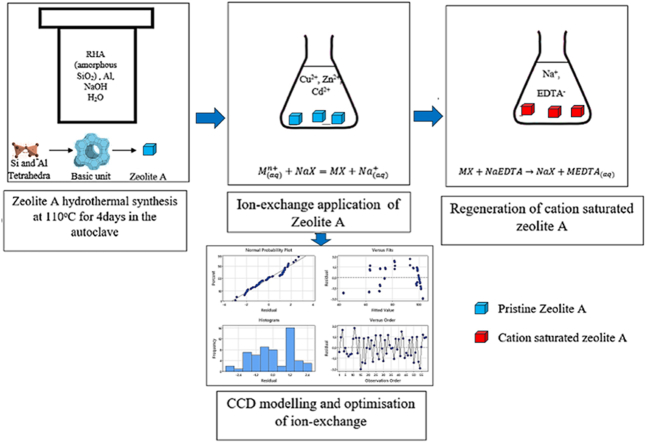

## 1 Introduction

For many decades, the pollution of the hydrosphere has always been considered one of the most vital issues worldwide, particularly in most developing countries [1]. Adjacent to the socio-economic growth resulting from mining, metallurgical, petroleum, textile, paper, and pharmaceutical industries, pollution of water resources remains a major drawback of the rapid increase of these numerous industries in most developing countries [[2], [3], [4]]. The common form of pollution of water resources is mainly linked to the presence of high concentrations of multi-cationic contaminants (i.e., Cu^2+^, Cd^2+^and Zn^2+,^ etc.) [5,6].

Over the years, the application of natural and synthetic zeolites for the purification of cationic contaminant-containing effluents has continued to attract the interest of numerous scientists and researchers as a result of the high ion exchange and sorption capacities of zeolites [7,8]. Synthetic and natural Zeolites are microporous crystalline hydrated tectosilicates mainly consisting of Si, Al, and O elements [9,10]. Zeolites generally consist of a three-dimensional framework of silicon [SiO_4_]^4−^ and aluminium [AlO_4_]^5−^ tetrahedra joined by the O^2−^ ions [11,12]. Zeolite lattices are negatively charged as a result of the substitution of Si^4+^ with Al^3+^. The negatively charged zeolite lattices are thus balanced with alkali and alkali earth metal counterions (i.e., K^+^, Cs^+^, Na^+^, and Ca^2+^) to form neutral zeolite compounds [13,14]. The zeolite counterions can be exchanged with the heavy metal ions as a result of the difference in electrostatic forces, ionic radius, and energy hydration between the heavy metal ions and the zeolite counterions [9,[15], [16], [17]]. In previous studies, the maximum ion-exchange capacities of zeolites are reported in the following order: Pb^2+^> Cu^2+^> Cd^2+^>Ni^2+^>Mn^2+^ [[15], [16], [17]].

Considering the attractive ion-exchange property of zeolites for water treatment applications, the research on the optimization of kinetic parameters affecting zeolite ion-exchange efficiency continues to be a matter of importance in recent years [18]. Particularly, for the removal of heavy metal ions from polluted effluents, numerous studies have utilized the one-factor-at-a-time factor (OFAT) approach to optimize kinetic parameters affecting zeolite ion exchange efficiency [17,[19], [20], [21]]. However, the OFAT approach is often not conclusive, and cannot provide a mathematical model that describes the interactions between the independent variables (i.e. kinetic parameters) and the response (i.e. removal efficiency) [22,23]. Additionally, the lack of randomization using OFAT approach can lead to biased inferences. One way of overcoming these limitations lies in the use of response surface methodology (RSM) [24].

**Fig. 1:**
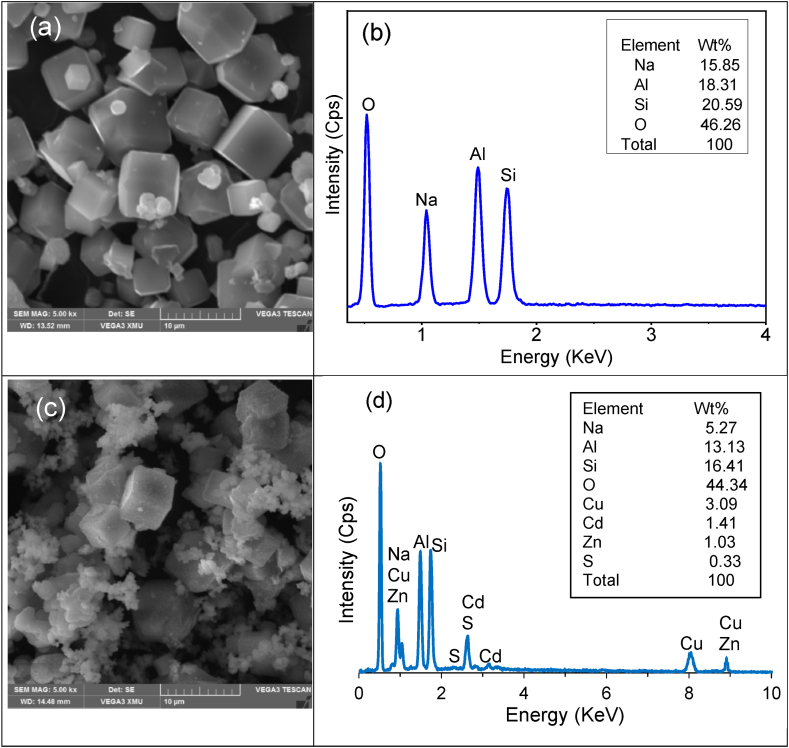
SEM micrographs: (a) Zeolite A before ion-exchange and (b) Zeolite after ion exchange and EDS spectrums: (c) Zeolite A before and (d) Zeolite after ion exchange.

RSM is a developed set of mathematical and statistical techniques for designing experiments, modeling, analyzing optimal conditions, and simultaneously evaluating the effects of independent variables on a given response [25]. RSM has been effectively used for the optimization of different processes such as adsorption, separation processes, chromatography [[26], [27], [28]]. The current study aims to model the metal ion removal efficiency of zeolite 4A as a function of key kinetic parameters, and then optimize those parameters using the central composite design (CCD) methodology from response surface methodology (RSM). This approach can provide a more comprehensive optimization of the zeolite ion exchange process compared to OFAT methods. Additionally, this work will provide insights into the underlying ion exchange mechanisms based on kinetic, thermodynamic, and isotherm analyses. The graphical abstract is shown in Fig. 1.

## 2 Materials and methods

### 2.1 Materials

The multi-cationic contaminant-based effluent with varied cationic concentrations was prepared using 99 % ZnSO_4_.H_2_O, CuSO_4_.5H_2_O, and CdCl_2_.H_2_O high-purity grade salts supplied by ACE Chemicals. 1M NH_4_Cl was prepared to determine the cation exchange capacity. Zeolite 4A was hydrothermally synthesized using rice husk (RH) ash and food-grade aluminium foil wastes. The combustion of rice husk at 600 °C produced ash rich in amorphous and reactive SiO_2_. Before combustion, rice husk was washed with 3 % HCl (solid mass to liquid volume ratio equivalent to 50 g/L) to upgrade the SiO_2_ content from 94 % up to 98 %. This allows the synthesis of zeolite with high purity.

### 2.2 Synthesis of Zeolite 4A

The method of synthesis of zeolite using waste materials as sources of silicon and food-grade aluminium foil is considered eco-friendly [11]. Synthesis of zeolite was carried out in an autoclave placed in the oven at 110 °C [9,29]. The zeolite crystallization time was 4 days. SiO_2_: Al_2_O_3_:Na_2_O: H_2_O molar ratios for the hydrothermal synthesis of Zeolite A were 1:0.83:1.15: 150 [9]. The quantities of reagents and precursors used in the current study are summarized in Table 1. After crystallization, the zeolite 4A was filtered from the alkaline solution, washed twice with 500 ml of deionized water, and dried at 50 °C in the oven.

**Table 1:** Quantities of reagents and precursors used for the synthesis of zeolite 4A.

Reagents and precursors	Quantity (g)
NaOH	31.46
RHA (amorphous SiO_2_)	22.7
Al waste foil	15.28
H_2_O	898.74

### 2.3 Experimental procedure of batch ion-exchange

Zeolite and multi-cationic contaminant effluent were mixed at varied pH, contacting time (hour), zeolite dosage (g/100 ml), and temperature (^o^C) using the Ecobath thermoshaker Sepsci model. The initial concentration of each selected cation is 450 mg/L. The stirring rate and the volume of the solution were maintained constant at 140 rpm and 100 ml. The investigated parameters are summarized in Table 2. The ion-exchange removal efficiency percent (R%) and ion exchange capacity (q) are determined according to Equations (1), (2)) respectively.

(1) $$R\left(\%\right)=100\;\frac{\;C_{i}-C_{f}}{C_{i}}$$

(2) $$q\;\left(mg.g^{-1}\right)=\left(C_{i}-C_{f}\right)V/m$$

where C_i_ and C_f_ represent the initial and final concentrations in mg. L^−1^ before and after ion exchange respectively. V and m respectively denote the volume (ml) of the multi-cationic contaminant-based effluent and the mass (g) of zeolite 4A. Zeolite 4A was mixed with contaminated effluent at a constant 140 rpm.

**Table 2:** Ion exchange parameters for the removal of Cd^2+^, Cu^2+,^ and Zn^2+^ cations.

Varied parameters	Range	Interval	Initial concentration (mg/L)
Contact time (hours)	1–5	1	450
Temperature (^o^C)	25–35	2.5	450
Zeolite dosage (g/100 ml)	0.4–1.2	0.2	450
pH	1–6	1	450

### 2.4 Regeneration of zeolite 4A

Na-EDTA solution was used as the eluent to regenerate the saturated zeolite 4A. Before regeneration, 1.2g of zeolite 4A was mixed with 100 ml of a solution containing Cd^2+^, Cu^2+,^ and Zn^2+^ cations. The initial concentration of each selected cation is 450 mg/L. The mixture was shaken for 24 h at 140 rpm and 25 °C. The step was repeated three times to ensure complete saturation of zeolite 4A. Subsequently, the saturated zeolite 4A was mixed with 100 ml of 0.05M Na-EDTA at 25 °C for 3 h. Lastly, the mixture was filtered, and the concentration of the solution after regeneration was determined using the atomic absorption spectrometric analytical technique.

### 2.5 Response surface methodology (RSM) design

Response surface methodology design of experiments (DoE) consisted of a central composite design (CCD) with 3 blocks, 2 axial points, 29 based experiments, and 2 replicates using Minitab software. The Minitab software interface automatically generated the design of the experiments in Table 3. The 29 experiments are presented in Table 3. The Cd^2+^, Cu^2+^, and Zn^2+^ removal efficiencies presented in Table 3 are calculated from the final cation concentrations of solutions after the simultaneous cation exchange using Zeolite 4 as per the DoE. The evaluation of full quadratic RSM models for the removal efficiency percentages of Cu%, Zn%, and Cd% as the responses was evaluated based on the coefficients of correlation R^2^ and adjusted R^2^. The effect of parameter terms in the model was evaluated based on their level of significance (p). The threshold p-level of significance was set at 0.05 [30,31]. The fitness of the model to the experimental data was also evaluated based on the relative linearity of normality plots. The generalized full quadratic response model y is given as [32]:

$$y=\beta_{o}+\beta_{1}X_{1}+\ldots .\beta_{k}X_{k}+\ldots .\beta_{11}^{2}X_{1}^{2}+\ldots \beta_{kk}^{2}X_{k}^{2}+\beta_{12}XX_{2}+\ldots .\beta_{k-1,k}X_{k-1}X_{k}$$

where β_0_, β_1 …_ β_k_ represent the variable coefficients. X_1_ … X_k_ represents the variable terms and k is the number of independent variables or parameters.

**Table 3:** Central composite design (CCD) of experiments and experimental cation removal efficiencies %.

Order	Ion exchange parameter				Experimental removal %		
	Time (hours)	Dosage (g/100 ml)	Temp (^o^C)	pH	Cd^2+^(%)	Cu^2+^(%)	Zn^2+^(%)
1	4	0.6	27.5	3	50.24	69.63	36.95
2	2	1	27.5	3	63.00	83.85	53.11
3	2	0.6	32.5	3	41.12	64.19	28.85
4	4	1	32.5	3	59.60	76.00	55.98
5	2	0.6	27.5	5	63.69	100	37.73
6	4	1	27.5	5	93.90	100	81.63
7	4	0.6	32.5	5	64.69	100	48.12
8	2	1	32.5	5	80.07	100	72.67
9	3	0.8	30	4	67.85	96.78	48.46
10	3	0.8	30	4	67.85	97.09	48.46
11	2	0.6	27.5	3	47.43	67.99	31.46
12	4	1	27.5	3	66.76	83.50	58.59
13	4	0.6	32.5	3	44.71	65.04	34.34
14	2	1	32.5	3	55.06	74.07	50.50
15	4	0.6	27.5	5	69.43	100	47.41
16	2	1	27.5	5	87.22	100	71.96
17	2	0.6	32.5	5	58.17	100	38.44
18	4	1	32.5	5	87.53	100	82.34
19	3	0.8	30	4	67.85	97.08	48.46
20	1	0.8	30	4	57.89	100	36.41
21	5	0.8	30	4	68.17	100	51.57
22	3	0.4	30	4	66.62	95.90	55.00
23	3	1.2	30	4	100	100	100
24	3	0.8	25	4	71.45	72.14	21.46
25	3	0.8	35	4	58.77	60.53	19.57
26	3	0.8	30	2	22.09	41.02	32.15
27	3	0.8	30	6	66.28	100	64.77
28	3	0.8	30	4	68.11	97.18	48.46
29	3	0.8	30	4	69.11	97.08	48.46

### 2.6 Characterization

Zeolite 4A and RHA were characterized through X-ray diffraction (XRD), X-ray fluorescence (XRF), scanning electron microscope (SEM), and Brunauer-Emmett-Teller (BET) analyses. The chemical compositions of RHA and zeolites were determined using a 4 kW WDXRF sequential-wavelength dispersive X-ray fluorescence spectrometer. The X-ray diffraction pattern of zeolite before and after ion exchange was studied using Rigaku Ultima IV X-ray spectrometers operating at 40 kV and 30 mA equipped with a cu- X-ray source (X-ray wavelength *λ* of 15.406 nm), a scan rate of 4/min, and step width of 0.02°. The morphology and chemical compositions of the zeolite and RHA were characterized at varied magnifications through a TESCAN Vega 3 XMU scanning electron microscope equipped with an energy dispersive spectrometer (EDS) operating at beam intensity and a high voltage of 20 kV and 11W/m^2^ in a secondary electron mode. Aliquots of solution concentrations of cationic contaminant-based effluent before and after ion exchange were determined using a Thermo Scientific Ice 300 Series atomic absorption spectrometer (AAS). Cd, Cu, and Zn wavelengths are 228.8 nm, 324.7 nm, and 213.9 nm, respectively. The evaluation of surface areas, pore size, and pore volume was carried out using a Tristar II Plus BET analyzer. The Microtrac Sizer and the Micromeritics AccuPyc II 1340 pycnometer were utilized for the analysis of the particle size distribution and the density of the synthesized zeolite.

## 3 Results and discussion

### 3.1 Characterization

#### 3.1.1 Evaluation of the elemental composition and morphology of zeolite before and after removal of Cd^2+^, Cu^2+^ and Zn^2+^ ions

**Fig. 2:**
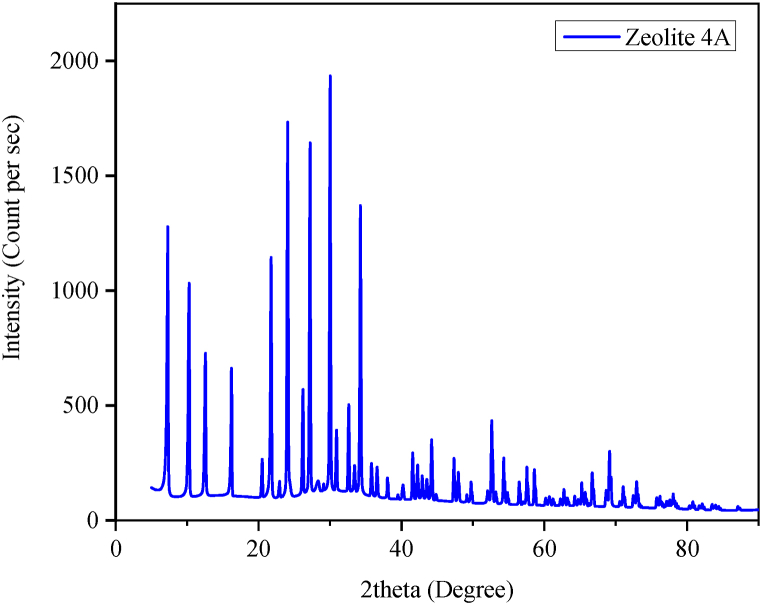
X-ray diffraction pattern of the synthesized zeolite 4A.

Based on the weight percentage elemental compositions of zeolite 4A in Table 4, the calculated molecular ratio of Na:Si:Al is approximately equivalent to 1 corresponding to the unit cell of Na_12_(SiO2)_12_(AlO_3_)_12_.nH_2_O. The synthesized zeolite can be classified as zeolite 4A. As a result of the high aluminium content and the substitution of Si with Al within the zeolite lattice, zeolite 4A thus possesses a high ion exchange capacity equivalent to 5.31 meq/g. Zeolite A ion exchange capacity and physical characteristics are presented in Table 5. The micrographs of the synthesized zeolite in Fig. 1 (a) and (c) show a cubic unit system that corresponds to the zeolite 4A phase. These results are also in good agreement with EDS analyses in Fig. 1(c) and (d) in the current study. This further corroborates XRD results in Fig. 2.

**Table 4:** Elemental compositions of zeolite before and after ion exchange.

Oxide (wt. %)										
Zeolite	Al_2_O_3_	CaO	Fe_2_O_3_	Na_2_O	K_2_O	SiO_2_	CdO	CuO	ZnO	Other
Before	29.56	0.05	1.33	18.1	0.15	41.26	–	–	–	0.38
After	26.55	0.01	1.01	7.14	0.02	44.5	15.55	4.1	1.02	0.10

**Table 5:** Zeolite A ion exchange capacity and physical characteristics.

Ion exchange capacity (mg/g)	5.31
BET Surface area (m^2^/g)	32.56
Pore size (nm)	12.45
Pore volume (cm^3^/g)	0.0087
Specific density (g/cm^3^)	2.56
Bulk density (g/cm^3^)	0.703
Particle size (μm)	−75

As expected, zeolite 4A exhibits a strong affinity for Cd^2+^, Cu^2+^, and Zn^2+^ ions from the synthetic effluent due to ion exchange with the counter ions of zeolite (Na^+^ ions). Hence, the results in Table 4 indicate a significant decrease in the amount of sodium Na from 18.1 % to 7.14 % after the removal of cationic contaminants. Previous studies have demonstrated that the counter ions of zeolite are electrostatically bound on zeolite 4A and can be exchanged with other cations [9,15,33]. In comparison with XRF results, the EDS results of zeolite 4A before and after ion exchange in Table 6 also indicate a significant decrease in Na amount from 15.85 % to 5.27 % due to the ion exchange with cationic contaminants.

**Table 6:** Energy dispersive spectrometric (EDS) analysis of zeolites before and after adsorption.

Element (wt. %)								
Zeolite	Al%	Cd%	Cu%	Na%	O%	S%	Si%	Zn%
Before	18.31	–	–	15.85	45.26	–	20.59	–
After	13.13	1.41	3.09	5.27	44.34	0.33	16.41	1.03

#### 3.1.2 X-ray analysis of the synthesized zeolite 4A

X-ray diffraction analysis in Fig. 2 further confirmed the formation of zeolite 4A. The synthesized zeolite major peaks diffract at 10.30, 12.56, 16.19, 20.47, 21.72, 24.05, 26.17, 27.17, 29.98, 30.87,32.59, 33.4 and 34.22. These 2-theta corroborate the findings of previous studies [9],^34^,^35^.

### 3.2 Effects of selected ion exchange parameters on Cd^2+^, Cu^2+^, and Zn^2+^ removal efficiencies

#### 3.2.1 Effects of contact time and dosage

**Fig. 3:**
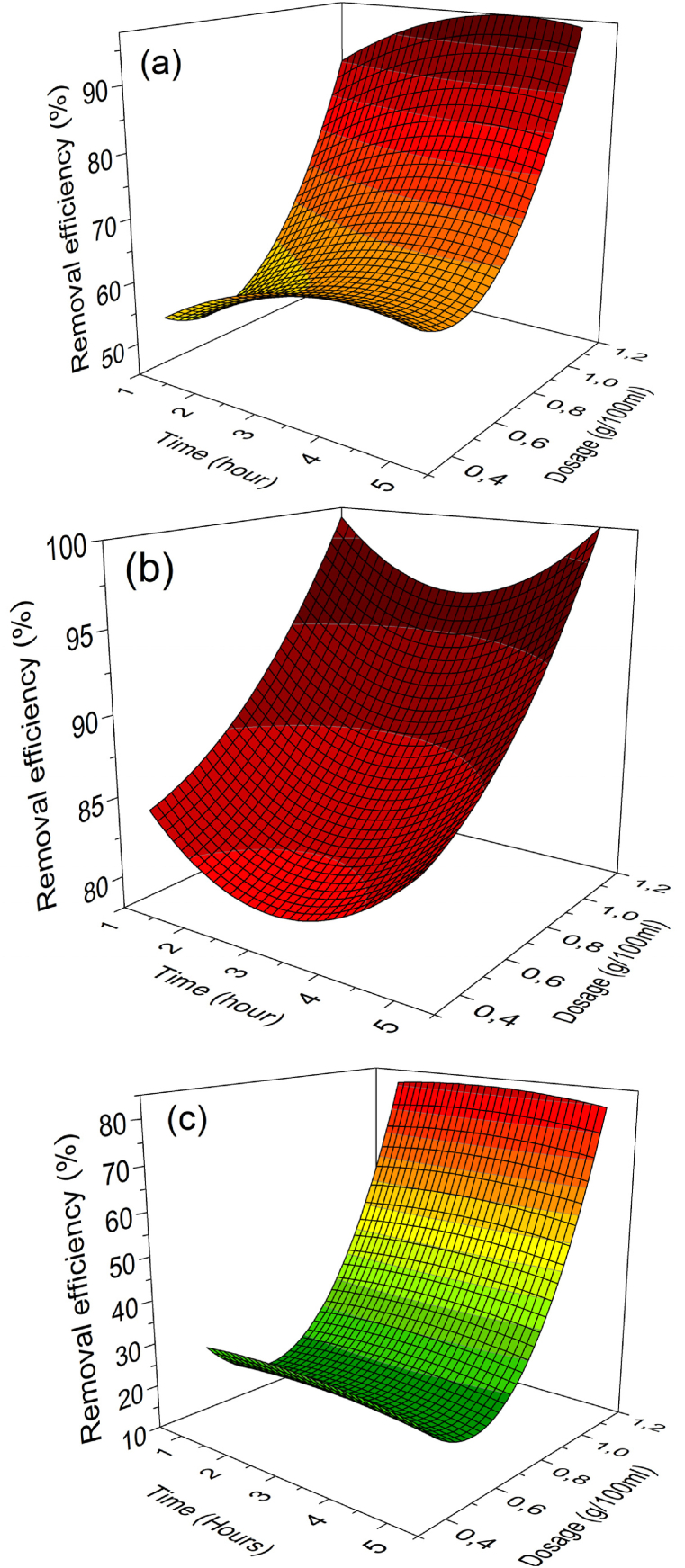
Response surface plots of removal efficiencies as a function of time (hour) and zeolite dosage (g/100 ml): (a) Cd^2+^, (b) Cu^2+^, and (c) Zn^2+^ ions.

In the current study, the effects of parameters are evaluated based on the surface response plots of the Cd^2+^, Cu^2+^, and Zn^2+^ removal efficiency models shown in Fig. 3(a–c) and 5 (a-c). Analysis of the variance of models in Table 5, Table 6, Table 7 has been carried out to evaluate the goodness of fit of the models-based CCD approach. Results in Fig. 3 indicate that ion exchange efficiency is time dependent. The results indicate a rapid initial ion exchange or sorption rate (more than 80 % of metal ion removal has been achieved within 1 h of the contact period with a zeolite dosage of 1.2 g/100 ml).

**Table 7:** Analysis of variance of Cd^2+^ removal efficiency regression model.

Source	DF	Seq SS	Contribution	Adj SS	Adj MS	F-Value	P-Value
Model	16	13845.5	99.83 %	13845.5	865.35	1523.66	<0.001
Blocks	5	2.4	0.02 %	3.9	0.77	1.36	0.026
Linear term	4	10610.8	76.51 %	10610.8	2652.69	4670.72	<0.001
A	1	345.7	2.49 %	345.7	345.68	608.65	<0.001
B	1	4044.7	29.16 %	4044.7	4044.67	7121.65	<0.001
C	1	474.9	3.42 %	474.9	474.9	836.17	<0.001
D	1	5745.5	41.43 %	5745.5	5745.51	10116.41	<0.001
Square term	4	3072.1	22.15 %	3072.1	768.03	1352.31	<0.001
A^2^	1	32.4	0.23 %	67	67.01	117.98	<0.001
B^2^	1	1272.9	9.18 %	729.2	729.19	1283.93	<0.001
C^2^	1	11.7	0.08 %	22.4	22.36	39.37	<0.001
D^2^	1	1755.2	12.66 %	1755.2	1755.21	3090.48	<0.001
2-Way Interaction	3	160.2	1.16 %	160.2	53.4	94.02	<0.001
AC	1	16.7	0.12 %	16.7	16.66	s	<0.001
BC	1	5.2	0.04 %	5.2	5.25	9.24	0.004
BD	1	138.3	1.00 %	138.3	138.28	243.47	<0.001
Error	41	23.3	0.17 %	23.3	0.57		
Lack-of-Fit	37	22.8	0.16 %	22.8	0.62	4.92	0.035
Pure Error	4	0.5	<0.01 %	0.5	0.13		
Total	57	13868.8	100.00 %				

*DF, SS, and MS denote the degree of freedom, the sum of squares and the mean square respectively.

This is justified by the high amount of exchangeable Na^+^ ions within the first hour. These results corroborate the findings of previous studies [36]. A further increase in time ensures the ion exchange reaches its equilibrium point. After 5 h, the removal efficiencies of Cd^2+^, Cu^2+^, and Zn^2+^ were reported as 96.5 %, 99.9 %, and 83.7 %. Cd^2+^, Cu^2+^, and Zn^2+^ uptake capacity values of zeolite 4A at time t (hours) were thus reported as 17.5 mg/g, 18.5 mg/g, and 15.69 mg/g.

**Fig. 4:**
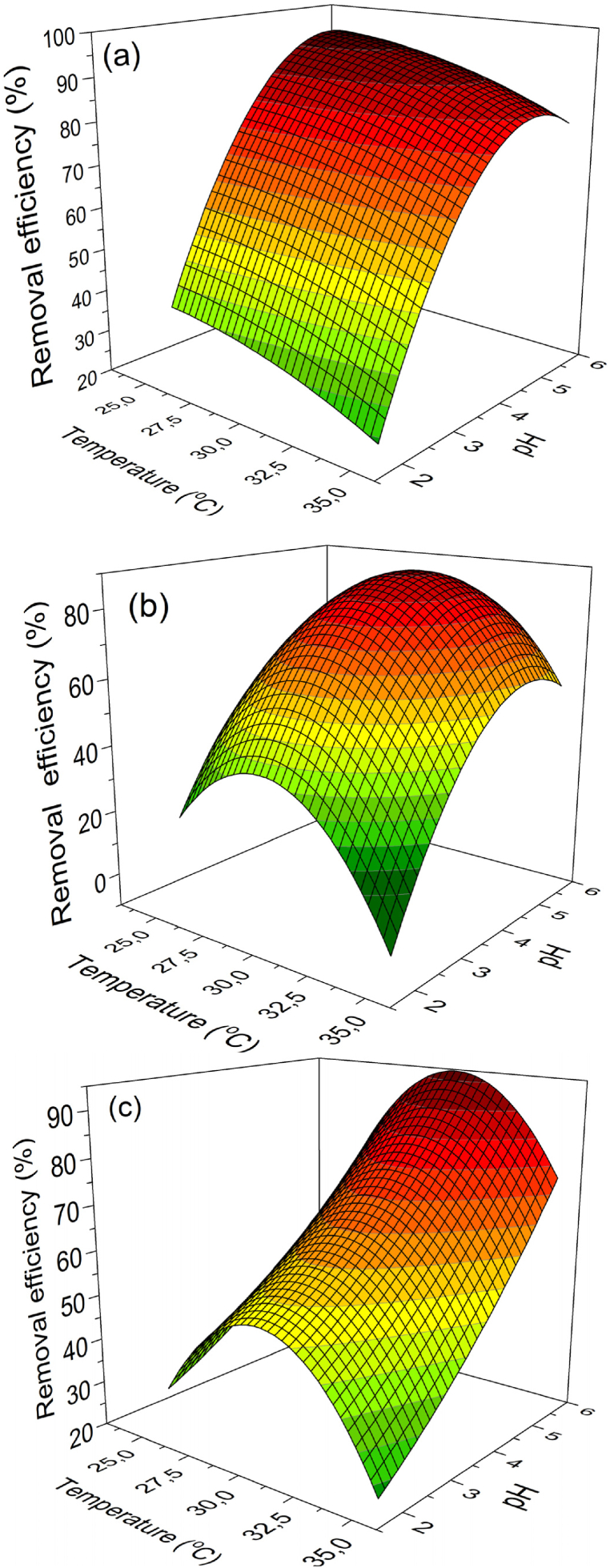
Response surface plots of removal efficiencies as a function of temperature (^o^C) and pH: (a) Cd^2+^, (b) Cu^2+^, and (c) Zn^2+^ ions.

On the other hand, Cd^2+^, Cu^2+^, and Zn^2+^ removal efficiency percentages significantly increase with the increase in zeolite dosage, as illustrated in Fig. 4. This could be expected because, with the increase in zeolite dosage in contact with the cationic contaminant-based effluent, the number of exchange sites (i.e. sites holding exchangeable Na^+^ ions) available for the electrostatic adsorption of cationic ions increases, thus more cationic contaminants can be exchanged with counter-ions [37,38].

#### 3.2.2 Temperature and pH effects

Response surface plots in Fig. 4 show that a higher temperature range above 30 °C hinders zeolite ion exchange; Cd^2+^, Cu^2+^, and Zn^2+^ removal efficiency percentages dropped from 94 % to 75 % when the temperature rose from 30 to 35 °C. This implies that zeolite ion exchange with cationic contaminants is exothermic. These results corroborate the findings of studies on the zeolite ion exchange of cationic contaminants [39,40].

**Fig. 5:**
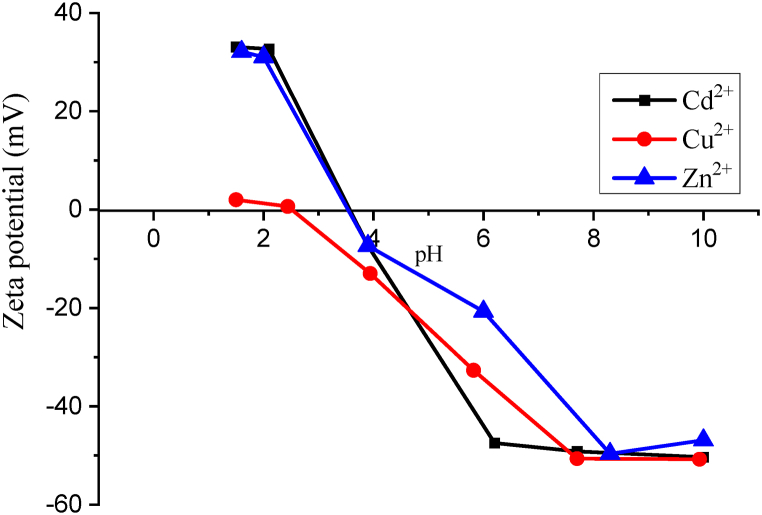
Zeta potential profile of zeolite 4A as a function of pH in the presence of varied metal ions.

One important parameter of ion exchange is the potential in H^+^ concentration (pH). Considering the ion-exchange process is mainly governed by the difference in electrostatic forces and zeta potential, pH as the ion exchange controls the zeolite surface charge of minerals [41]. Cd^2+^, Cu^2+^, and Zn^2+^ removal efficiency percentages were above 90 % at a pH range of 4–6 as illustrated in Fig. 4. This is justified by the fact that generally, the increase in pH just above the dielectric point pH_z_ (pH > pHz) tends to render the surface of the hydrophilic zeolite surface more negative and thus able to attract Cd^2+^, Cu^2+^, and Zn^2+^ through columbic forces. In Fig. 5, the dielectric points pHz of Cd^2+^ and Zn^2+^ are reported approximately equivalent to 3.5 and that of Cu^2+^ equivalent to 2.7.

The ion exchange efficiency also depends on the speciation of metal ions. with the increase in pH up to 5.7, Cu ions are mainly in the form of Cu (OH)^+^ whilst Cd^2+^ and Zn^2+^ mainly remain as free divalent ions in an aqueous system. However, the removal of metals with a further increase above neutral pH essentially takes place by the chemical precipitation of metals as hydroxides rather than an ion exchange process. For the complete chemical precipitation of Cd^2+^, Cu^2+^, and Zn^2+^ cations from aqueous solutions, pH values are 10.5, 8, and 10 respectively [42].

On the other hand, zeolite surface in a slightly acidic media (1<pH < 4), zeolite tends to be positively charged due to the protonation of its zeolite. The protonation of the silicate surface is given in Equation (3). There is also a competition between H_3_O^+^ ions and the metal cations. As illustrated in Fig. 5, the positive Zeta potential values of cations confirm that the surface of zeolite is positively charged. With a positively charged surface, cations are not attracted toward zeolite particles as a result of electrostatic repulsion forces. This possibly justifies the low removal efficiency in acidic media.

(3) $$Si{\left(OH\right)}_{3}O^{-}+2H_{2}O\to Si\;{\left(OH\right)}_{4}H^{+}+OH^{-}$$

However, zeolite A becomes unstable and decomposes at low pH < 1 due to the dealumination. Previous studies stipulate that high metal ion removal efficiency by ion exchange using zeolite is generally obtained at a pH ranging from 4 to 6 [33,43].

### 3.3 Analysis of variance (ANOVA) of the removal efficiency models

In the current study, the ANOVA has been used to evaluate the goodness of fit of CCD models to the experimental ion exchange. The Minitab software interface processes the response as a function of the kinetic parameters and further provides ANOVA data and the quadratic models presented in this study. In the current study, removal efficiency is the response. The analysis of variance in Table 5, Table 6, Table 7 indicates a strong relationship between removal efficiency models and the experimental data with the lack of fit and model error percentages for all the models being less than 1 % [44].

**Fig. 6:**
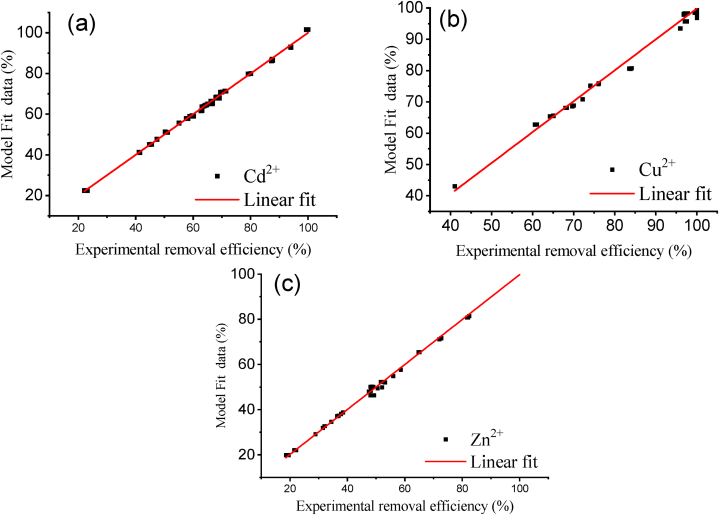
Plots of Experimental removal efficiency versus model fit data: (a) Cd^2+^, (b) Cu^2+^ and (c) Zn^2+^.

**Fig. 7:**
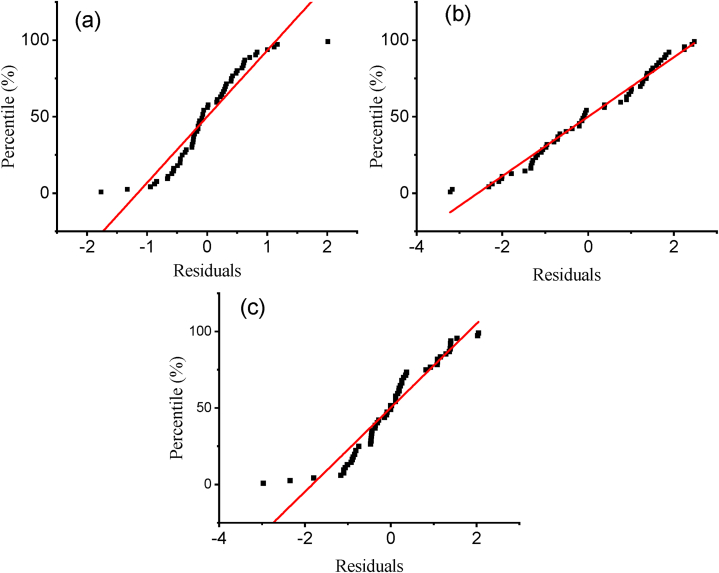
Normality probability plots of residuals*:* (a) Cd^2+^, (b) Cu^2+^ and (c) Zn^2+^.

The respective correlation coefficients R^2^ for Cd^2+^, Cu^2+^, and Zn^2+^ removal efficiency models were reported as 0.9983, 0.9917, and 0.9953. The adjusted R^2^ for Cd^2+^, Cu^2+^, and Zn^2+^ are equivalent to 0.9891, 0.9978, and 0.9934. The linearity of plots of experimental removal efficiency versus the model data in Fig. 6 further confirms the goodness of fit of the generated models based on CCD methods. The normal probability plots of the residuals from the model fit and the experimental data in Fig. 7 are reasonably linear; they thus, suggest that the error randomness is acceptable. Therefore, the optimal conditions of the ion exchange process for the removal of metal ions can be determined based on these models with satisfactory desirability.

Results in Table 6, Table 7, Table 8 also indicate that all the effects of the evaluated parameters are statistically significant with their level of significance less than the set threshold p < 0.05) [30,31]. Cd^2+^, Cu^2+^ and Zn^2+^ cumulative removal efficiencies respectively increased from 90 % to 96 %, 98 %–99.97 % and 80 %–85 %. The Cd^2+^, Cu^2+^ and Zn^2+^ removal efficiency models are given in Equations 2, 3 and 4.

(2) $${Cd}^{2+}\;R\%=-98.3+6.631A-121.68B+5.71C+47.10D-1.139A^{2}+93.94B^{2}-0.1053C^{2}-5.830D^{2}-0.722AD-0.810BC+10.394BD$$

(3) $$Cu^{2+}\;R\%=-1035.7-6.82A+48.4B+65.95C+56.99D+1.1629A^{2}+16.39B^{2}-1.1556C^{2}\;-6.247D^{2}-15.64BD+0.645CD$$

(4) $$Zn^{2+}\;R\%=-724.4+2.03A-298.06B+59.32C-22.86D-0.410A^{2}+188.32B^{2}-1.0140C^{2}+0.682D^{2}-1.060A+15.81+0.326CD$$

where A, B, C and D terms represent the contacting time (h), zeolite dosage (g/100 ml), temperature (^o^C) and pH, respectively as variables of removal efficiency R%.

**Table 8:** Analysis of variance of Cu^2+^ removal efficiency regression model.

Model	DF	Seq SS	Contribution	Adj SS	Adj MS	F-Value	P-Value
Model	15	15025.5	99.17 %	15025.5	1001.7	335.06	<0.001
Blocks	5	61.5	0.41 %	71.6	14.31	4.79	0.001
Linear term	4	9679.7	63.89 %	9679.7	2419.93	809.43	<0.001
A	1	1.1	0.01 %	1.1	1.14	0.38	0.050
B	1	281.8	1.86 %	281.8	281.8	94.26	<0.001
C	1	196.3	1.30 %	196.3	196.33	65.67	<0.001
D	1	9200.4	60.72 %	9200.4	9200.43	3077.42	<0.001
Square term	4	4888	32.26 %	4888	1222	408.74	<0.001
A^2^	1	487.6	3.22 %	69.7	69.74	23.33	<0.001
B^2^	1	442.6	2.92 %	22.2	22.21	7.43	0.009
C^2^	1	1942.3	12.82 %	2694.3	2694.3	901.21	<0.001
D^2^	1	2015.5	13.30 %	2015.5	2015.45	674.14	<0.001
2-Way Interaction	2	396.3	2.62 %	396.3	198.14	66.28	<0.001
BD	1	313.1	2.07 %	313.1	313.14	104.74	<0.001
CD	1	83.1	0.55 %	83.1	83.14	27.81	<0.001
Error	42	125.6	0.83 %	125.6	2.99		
Lack-of-Fit	38	125.1	0.83 %	125.1	3.29	28.16	0.003
Pure Error	4	0.5	<0.01 %	0.5	0.12		
Total	57	15151.1	100.00 %				

*DF, SS, and MS denote the degree of freedom, the sum of squares and the mean square respectively.

Furthermore, the results in Table 7, Table 8, Table 9 also stipulate that, based on the contribution to the model, the zeolite dosage, temperature, and pH parameters are the most important effects. Contribution percentages of time to the models or removal efficiency regression function are significantly lower when compared to the contribution percentage of the zeolite dosage (g) in particular. The cumulative time contribution percentages of linear and square terms to the correlation coefficient of Cd^2+^, Cu^2+^, and Zn^2+^ removal efficiency models were respectively reported as 2.72 %, 3.23 %, and 3.73 %.

**Table 9:** Analysis of variance of Zn^2+^ removal efficiency regression model.

Source	DF	Seq SS	Contribution	Adj SS	Adj MS	F-Value	P-Value
Model	16	19214.4	99.53 %	19214.4	1200.9	537.6	<0.001
Blocks	5	192.9	1.00 %	185.8	37.16	16.64	<0.001
Linear term	4	12418.4	64.32 %	12418.4	3104.6	1389.81	<0.001
A	1	695.5	3.60 %	695.5	695.5	311.35	<0.001
B	1	8487.4	43.96 %	8487.4	8487.36	3799.47	<0.001
C	1	14.6	0.08 %	14.6	14.55	6.52	0.015
D	1	3221	16.68 %	3221	3221	1441.92	<0.001
Square term	4	6226	32.25 %	6226	1556.5	696.79	<0.001
A^2^	1	25.3	0.13 %	8.7	8.68	3.89	0.045
B^2^	1	3931.3	20.36 %	2930.8	2930.75	1311.99	<0.001
C^2^	1	2245.3	11.63 %	2074.6	2074.62	928.73	<0.001
D^2^	1	24	0.12 %	24	24.04	10.76	0.002
2-Way Interaction	3	377.1	1.95 %	377.1	125.68	56.26	<0.001
AD	1	36	0.19 %	36	35.96	16.1	<0.001
BD	1	319.9	1.66 %	319.9	319.89	143.2	<0.001
CD	1	21.2	0.11 %	21.2	21.2	9.49	0.004
Error	41	91.6	0.47 %	91.6	2.23		
Lack-of-Fit	37	90.7	0.47 %	90.7	2.45	10.59	0.016
Pure Error	4	0.9	<0.01 %	0.9	0.23		
Total	57	19306	100.00 %				

*DF, SS and MS denote the degree of freedom, the sum of squares and the mean square respectively.

### 3.4 Removal efficiency optimization

**Fig. 8:**
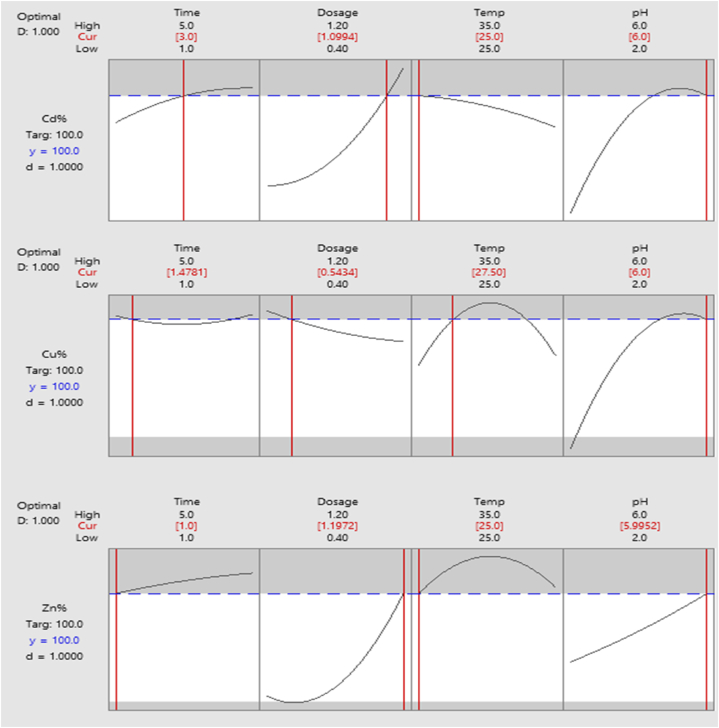
RSM Removal efficiency optimization plots.

Results from RSM optimization of selected kinetic parameters are shown in Fig. 8. The initial concentration of each selected cation was 450 mg/L. The optimal time range for the complete simultaneous removal of all cationic contaminants is from 1 to 3 h. The optimal dosage values for the complete removal of Cd^2+^, Cu^2+^, and Zn^2+^ were equivalent to 1.2 g/100 ml respectively, at temperatures ranging from 25 °C to 27.5 °C. The high removal efficiency was obtained at pH 6. The optimization desirability values were equivalent to 1 (D = 1.000).

### 3.5 Isotherms

**Fig. 9:**
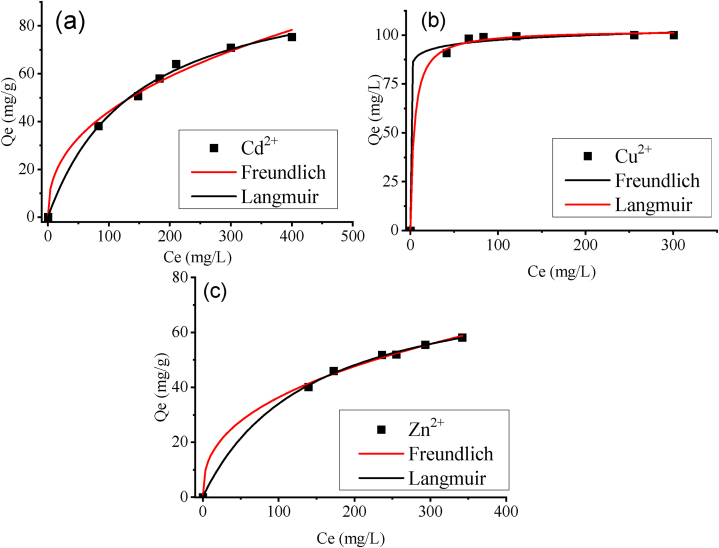
Langmuir and Freundlich isotherm plot at 25 °C: (a) Cd^2+^, (b) Cu^2+^ and (c) Zn^2+^.

Isotherms describe the underlying mechanisms between zeolite cations (the solutes) and zeolite 4A (the ion exchanger). Information on the maximum synthesized zeolite 4A adsorption or ion-exchange capacity Q_m_ has been obtained from the Langmuir model in Fig. 9 to evaluate the performance of zeolite as an ionic exchanger.

(5) $$q_{e}=K_{F}C_{e}^{\frac{1}{n}}$$

(6) $$q_{e}=\frac{{q_{m}K}_{L}C_{e}}{1+{K_{L}Q}_{e}}$$

where K_F_ and K_L_ denote Langmuir and Freundlich constants respectively. 1/n is an empirical constant related to the heterogeneity of the adsorbent surface.

The non-linear mathematical expressions of selected isotherm models are given in Equations 5 and 6. Based on the evaluation of correlation coefficients R^2^, results indicate that the Cd^2+^, Cu^2+^, and Zn^2+^ isotherms are in good agreement with the Langmuir and Freundlich models with R^2^ approaching unity (R^2^ > 0.9). The Langmuir model suggests that there is no interaction amongst the solutes that form a monolayer on the sorbent surface, and the energies of all the sorption sites on the sorbents are equivalent. However, such an assumption is not realistic. On the other hand, the Freundlich isotherm model stipulates that adsorbed species can form a heterogeneous multilayer, and the energies of the identical sorption sites are not evenly distributed or equal.

Langmuir models provide maximum ion-exchange capacity values for the cationic contaminants in the descending order of Cd^2+^>Cu^2+^>Zn^2+^. Adsorption constants, maximum ion exchange capacities, and correlation ratios are presented in Table 10. The selectivity of one cation over another is possibly attributed to differences in the energy of hydration, and electronegativity and the cation radius size. Previous studies by Fan et al. (2021) stipulate that Zeolite A has a high selectivity for metal ions with a lower energy of hydration in an aqueous solution. Metal ions in aqueous media become hydrated. The hydration diameter sizes of Cd^2+^, Cu^2+^ and Zn^2+^ are 0.852, 0.738 and 0.826 nm. Fan et al. (2021) suggest the hydrated Cd2+, Cu^2+^, and Zn^2+^ diameter sizes are bigger than the supercage of Zeolite A (0.74–0.78 nm). However, the lower the energy of cation hydration the more feasible the dehydration of the cation is. Consequently, the radius of the hydrated cation reduces after dehydration, and the cation can penetrate the supercage of Zeolite A. Cd^2+^ has the lowest energy of hydration equivalent to 1828 kJ/mol compared to those of Cu^2+^ and Zn^2+^ equivalent to 2121 and 2058 kJ/mol, respectively. This possibly justifies the higher uptake capacity of Cd^2+^ compared to Cu^2+^ and Zn^2+^. However, in the case of Zn^2+^ and Cu^2+^, although the energy of Zn^2+^ is lower than that of Cu^2+^, the high selectivity for Cu^2+^ when compared to Zn^2+^ is possibly justified by their difference in electronegativity. The Pauling electronegativity of Cu^2+^ (1.9) is higher than that of Zn^2+^ (1.65) [21].

**Table 10:** Isotherm constants and coefficients.

Cations	Freundlich			Langmuir		
	K_F_(mg.L^1/n^/g.mg^1/n^)	1/n	R^2^	Q_m_(mg/g)	K_L_(L/mg)	R^2^
Cd^2+^	24.8199	0.0208	0.9468	103.09	6.92x10^−3^	0.9965
Cu^2+^	83.01	0.306	0.9962	102.62	0.230	0.9983
Zn^2+^	2.1399	0.3904	0.9979	82.08	7.1 x10^−3^	0.9989

The summary of the comparative study of zeolite 4A with different adsorbents based on their sorption capacity is presented in Table 11.

**Table 11:** Comparative study between the synthesized zeolite 4A and other adsorbents.

Adsorbent	M^n +^ ions	Uptake capacity (mg/g)	Sorbent dosage(g/100 ml)	Initial concentration(mg/L)	pH	References
Zeolite 4A	Cd^2+^	103.09	1.2	450	6	This paper
	Cu^2+^	102.62	1.2	450	6	
	Zn^2+^	82.08	1.2	450	6	
Modified coal ash	Cu^2+^	96	2.5	100	5	[45]
Polysulfone/Zeolite blend	Cu^2+^	101	–	5–100	6	[46]
Zeolite from coal ash	Zn^2+^	177	0.1	300	5.5–6	[47]
Zeolite X	Cu^2+^	146	0.1	350	4.88	[48]
Zeolite X	Zn^2+^	195	0.1	350	5.48	[48]
NaP zeolite	Cd^2+^	117.3	2.5	100	5.2	[49]
Zeolite LTA	Cd^2+^	223.5	2.5	100	5.2	[49]

### 3.6 Thermodynamics

**Fig. 10:**
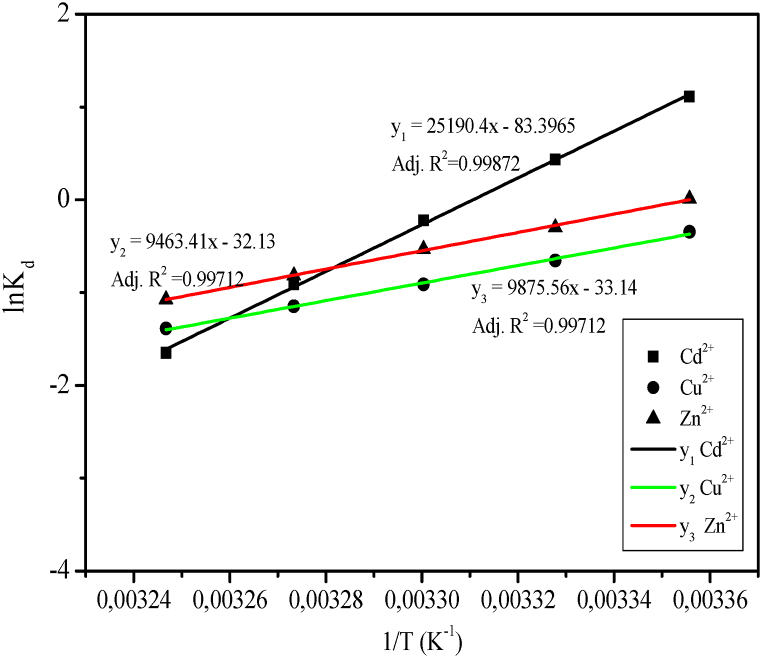
Linear plots of ln K_d_ versus 1/T.

.In Table 12, the difference in enthalpy ΔH and the entropy ΔS values were respectively calculated using the slope and the intercept of linear plots of ln K_d_ versus 1/T in Fig. 10. The mathematical expressions of ΔH, ΔS and ΔG are given in Equations 5, 6, and 7; where K_d_ and R respectively represent the ion exchange distribution coefficient and gas constants.

(5) $$K_{d}=\frac{Q_{e\;}}{C_{e}}$$

(6) ΔG=ΔH−TΔS

(7) $$\Delta G=-RTlnK_{d}$$

**Table 12:** Thermodynamic parameters.

Cations	ΔH^0^ (kJ.mol^−1^)	ΔS^0^ (kJ.mol^−1^.K^−1^)	ΔG^0^ (kJ.mol^−1^)
Cd^2+^	−209.432	−0.693	−415.946
Cu^2+^	−78.678	−0.267	−158.244
Zn^2+^	−82.104	−0.275	−164.054

Hence, the linear relationship of ion exchange equilibrium, enthalpy and entropy as a function of temperature is given as:

(8) $$\ln\;K_{d}=-\frac{\Delta H}{R}\;\left(\frac{1}{T}\right)+\frac{\Delta S}{R}$$

Hence, the linear relationship of ion exchange equilibrium, enthalpy and entropy as a function of temperature is given as:

(9) $$\ln\;K_{d}=-\frac{\Delta H}{R}\;\left(\frac{1}{T}\right)+\frac{\Delta S}{R}$$

The negative standard free energy ΔG^0^ implies that the sorption of Cd^2+^, Cu^2+^, and Zn^2+^ on the synthesized zeolite is thermodynamically feasible at 25 °C and spontaneous. On the other hand, the negative enthalpies ΔH^0^ stipulate that the sorption of Cd^2+^, Cu^2+^, and Zn^2+^ on the synthesized zeolite can be considered exothermic. The negative entropy ΔS^0^ is justified by the decrease in randomness of the system. The entropy of cations in the aqueous is lower than the entropy of cations sorbed by zeolite A. Consequently, the change in entropy is negative.

### 3.7 Reuse and regeneration of the eco-friendly synthesized zeolite 4A

**Fig. 11:**
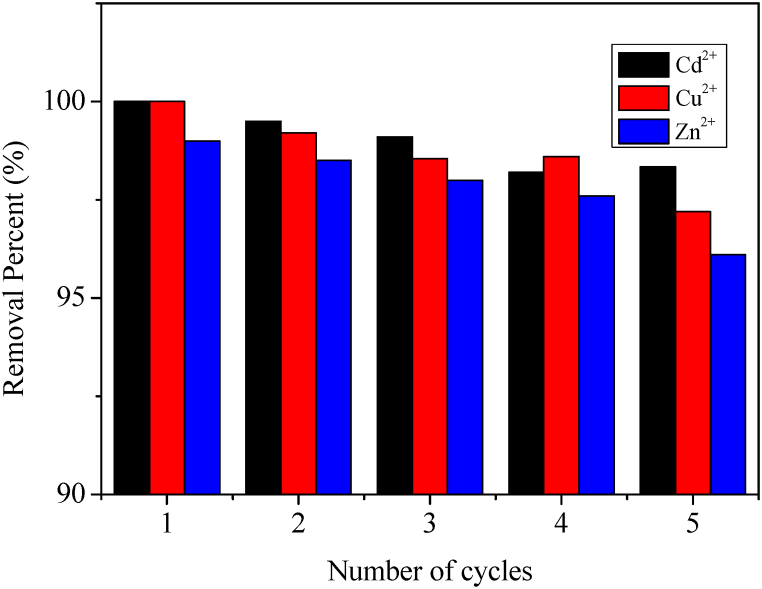
Reuse and regeneration application of zeolite 4A

The results in Fig. 11 stipulate that zeolite 4A can be regenerated using 0.05 M Na-EDTA. EDTA has the potential to regenerate Zeolite 4A due to its chelating property of metal ions. Thus, the adsorbed cationic contaminants are desorbed from the Zeolite 4A and solubilized in the eluent. However, the removal efficiencies of Cd^.2+^, Cu^2+^, and Zn^2+^ decrease with the number of cycles. This is possibly justified by the fact that the exchanged cations are not completely desorbed from zeolite 4A. Consequently, active sites of zeolite 4A are saturated as the number of cycles increases.

## 4 Conclusion

In the current study, a fundamental evaluation and optimization of the effects of dosage, time, temperature, and pH on the removal of Cd^2+^, Cu^2+^, and Zn^2+^ has been carried out using the RSM statistical approach. A strong relationship between the experimental data and the RSM models’ data has been observed, with an adjusted correlation coefficient approaching unity and a lack of fit less than 0.1 %. The optimal zeolite dosage and pH are 1.2 g/100 cm^3^ and 6, respectively. The optimal contact time varies from 1 to 3 h. The ion exchange system is maintained within the optimal temperature range of 25–27.5 °C. At the optimal conditions, 100 % removal of Cd^2+^, Cu^2+^, and Zn^2+^ was achieved with desirability equivalent to 1. CCD can thus be considered a useful tool for the modeling and optimization of zeolite ion exchange applications. In the current study, it has also been inferred that.

• Based on the contribution to the model, the zeolite dosage, temperature, and pH parameters are the most important effects, having the highest cumulative contribution percentage to the correlation coefficients of the models.
• As the pH increases, the cationic removal efficiency increases. The increase in pH just above the pH_z_ (pH > pHz) tends to render the surface of the hydrophilic zeolite surface more negative and thus able to attract Cd^2+^, Cu^2+^, and Zn^2+^.
• Since ion-exchange of Cd^2+^, Cu^2+^, and Zn^2+^ is favored at low temperatures. The zeolite ion exchange reaction for the removal of cationic contaminants is exothermic and possibly takes place by physisorption.
• Based on the evaluation of correlation coefficients R^2^, results indicate that the Cd^2+^, Cu^2+^, and Zn^2+^ isotherms are in good agreement with the Langmuir and Freundlich models, with R^2^ approaching unity (R^2^ > 0.9). The maximum Cd^2+^, Cu^2+,^ and Zn^2+^ uptake capacity values of zeolite are 103, 99.89, and 82.08 mg/g, respectively.

## Data availability

The data presented will be available on request from the corresponding author.

## CRediT authorship contribution statement

**Joseph M. Nseke:** Writing – review & editing, Writing – original draft, Software, Investigation, Conceptualization. **Nomsa P. Baloyi:** Writing – review & editing, Supervision, Project administration, Methodology, Conceptualization.

## Declaration of competing interest

The authors declare that they have no known competing financial interests or personal relationships that could have appeared to influence the work reported in this paper
